# Aesthetic Enhancement in Vertical Maxillary Excess by a Combined Orthodontic and Surgical Approach

**DOI:** 10.7759/cureus.75637

**Published:** 2024-12-13

**Authors:** Nikita Mohelay, Puneet Batra, Ashish K Singh

**Affiliations:** 1 Orthodontics and Dentofacial Orthopedics, Manav Rachna Dental College, Manav Rachna International Institute of Research and Studies, Faridabad, IND

**Keywords:** anterior maxillary osteotomy, genioplasty, le fort i osteotomy, orthognathic surgery, vertical maxillary excess

## Abstract

Vertical maxillary excess (VME) is a facial condition characterized by an increased height in the lower third of the face, leading to a longer overall facial appearance. This condition is linked to a significant proportion of malocclusions and is often associated with greater dissatisfaction among patients concerning their appearance. The amalgamation of orthodontics with surgery is a desirable protocol to address VME. The present clinical case exemplifies the effect of LeFort I with segmental anterior maxillary osteotomy and advancement genioplasty on improving the patient's skeletal, dental, soft tissue, and overall aesthetics.

## Introduction

Adults with severe skeletal malrelationships usually require an amalgamation of orthodontics and orthognathic surgical treatment to achieve optimum facial contours. The envelope of discrepancy indicates an orthodontic camouflage or orthognathic surgery in adults for addressing severe skeletal and facial disharmony. The orthodontic camouflage is difficult because jaw growth has terminated, and there is a higher likelihood of relapse following the correction [1]. Vertical maxillary excess (VME) is a facial abnormality characterized by an increased total anterior facial height resulting from an excessive vertical dimension in the lower third of the face. The prevalence of long face pattern seeking orthognathic-surgical treatment is approximately 22% [2].

VME presents with an excessive gummy smile, with increased overjet and a deep overbite. Gummy exposure of more than 3 mm is generally considered unattractive [3]. It is associated with higher levels of dissatisfaction in patients regarding appearance and has the potential to negatively impact their emotional and social well-being [4].

The surgical orthodontic treatment of VME using a LeFort I osteotomy is considered an ideal approach due to its benefits in skeletal stability and aesthetic soft tissue improvements, with the goal of minimizing negative impacts on health-related quality of life [5]. 

This clinical case typically exemplifies the effect of LeFort I with a segmental anterior maxillary osteotomy on improving the patient's skeletal, dental, soft tissue, and overall aesthetics.

## Case presentation

**Figure 1:**
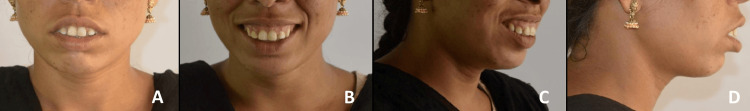
Pre-treatment extra-oral photographs. A: Frontal view; B: Frontal smiling view; C: Oblique smiling view; D: Profile view

An 18-year-old female patient presented with chief complaints of forwardly placed upper front teeth and an unattractive smile owing to the excessive gum display on smiling. There was no contributory medical or dental history. The extraoral facial analysis revealed a leptoprosopic facial form with an acute nasolabial angle, deep mentolabial sulcus, protruding upper and lower lips, and a receding chin. The face was grossly symmetrical with increased lower anterior face height proportions, as observed on frontal examination (Figure 1). On rest, the upper lip was incompetent with an incisor show of 10 mm, and on a smile, a consonant arc was observed, exhibiting complete incisor show along with 5 mm of gingival exposure.

Functionally, the patient was an oronasal breather. She presented with a hypotonic upper lip and hypertonic mentalis, a matching centric relation to centric occlusion with an aggravating soft tissue profile. She had a straight path of closure and showed no signs or symptoms of temporomandibular disorder.

**Figure 2:**
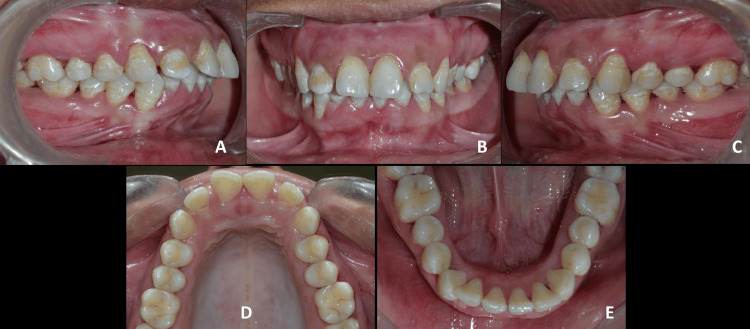
Pre-treatment intra-oral photographs. A: Right occlusion; B: Anterior in occlusion; C: Left occlusion; D: Maxillary arch; E: Mandibular arch

The intraoral examination showed that all permanent teeth, including the third molars, had erupted, with mild Grade I fluorosis present. She exhibited an Angle’s Class I molar relationship bilaterally and a Class II incisor relationship, with an overjet and overbite of 7 mm and 5 mm, respectively. The arch forms of both the maxilla and mandible were ovoid. The upper and lower dental midlines were aligned. Additionally, she had rotations in several teeth, along with mild crowding in the lower anterior region and mild gingivitis. The size and shape of the tongue were normal (Figure 2). 

**Figure 3:**
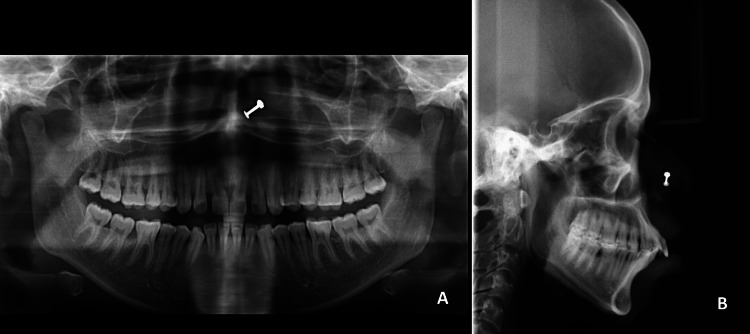
Pre-treatment radiographs. A: Orthopantomogram; B: Lateral cephalogram

Pre-treatment cephalometric examination indicated a skeletal class II (ANB, 8°) with prognathic maxilla (SNA, 88°) and Wits appraisal of 5 mm with hyperdivergent growth pattern (SN-MP, 35°; basal plane angle, 34°) and increased lower anterior facial height (LAFH) of 68 mm. The maxillary and mandibular incisors were proclined and protruded (U1 to NA, 35°, 12 mm)/(L1 to NB, 39°, 14 mm). A maxillary excess in vertical dimension was noted in the anterior and posterior dentition (U1-PP, 36.9 mm; U6-PP, 27.9 mm). At rest, there was relatively excessive exposure of the maxillary central incisors (U1 to stomion, 10 mm). The angle of the occlusal plane was within the normal range (OP to FH, 12.8°). The soft tissue analysis revealed an aggravating soft tissue profile, with a lower lip placed 13 mm ahead of the E plane and a reduced nasolabial angle at 83° (Figure 3; Table 1).

**Table 1:** Pre-treatment cephalometric summary

Variable	Pre-treatment
Sagittal skeletal relationship	
SNA	88⁰
SNB	80⁰
ANB	8⁰
Wits appraisal	5 mm
Dental base relationship	
Upper incisor to NA	12 mm/35⁰
Lower incisor to NB	14 mm/ 39⁰
Upper incisor to SN plane	120⁰
U1-NF/U6-PP	36.9 mm/27.9 mm
Lower incisor to mandibular plane angle	102⁰
Dental relationship	
Interincisal angle	101⁰
Lower incisor to A-Pog line	12 mm
Overbite	5 mm
Over jet	7 mm
Vertical skeletal relationship	
Maxillary mandibular plane angle	34⁰
SN plane-mandibular plane	35⁰
Upper anterior face height	42 mm
Lower anterior face height	68 mm
Face height ratio	38.1:61.9
Jarabak ratio	62.5%
Maxillary length (ANS-PNS)	57.5 mm
Effective mandibular length	107 mm
Soft tissue	
Lower lip-E plane	13 mm
Nasolabial angle	83⁰

Treatment Objectives

The treatment was designed to (a) establish an improvement in the position of the maxilla with (b) minimizing the gingival display on smile and at rest position, (c) to promote the autorotation of the mandibular arch, (d) harmonize the skeletal bases, (e) attain a skeletal class I relation, (f) level and align the arches, (g) achieve an ideal overbite and overjet, (h) address the lip incompetency, and (i) achieve an aesthetic, pleasant profile.

Treatment Alternatives

In order to address the VME in the patient, two treatment alternatives were considered: (1) Orthognathic surgery to efficiently deal with the increased maxillary height and protrusion via superior impaction and posterior setback of the maxilla. Additionally, favorable facial profile changes can be established by counter-clockwise rotation of the mandible accompanied by advancement genioplasty to address the chin deficiency. (2) Conventional orthodontics with temporary anchorage devices (TADs) for complete dental intrusion and retraction following the extraction of first premolars in the upper and lower arch. To address the gummy smile, intrusion of the entire maxillary dentition is needed, and intrusion of mandibular incisors and first molars to achieve counterclockwise rotation would also be required. The patient chose the surgical treatment plan.

Treatment Progress

The treatment began with a pre-adjusted edgewise appliance 0.022” × 0.028” slot (McLaughlin, Bennett, and Trevisi system, Ortho Organizers, Carlsland, CA, USA) and molar banding in both the arches. A continuous 0.016” nickel-titanium maxillary arch wire was initially inserted, which was progressively increased until 0.019 × 0.025” stainless steel wires. The mandibular first premolars were extracted, and initial alignment was initiated with a 0.016” nickel-titanium wire in the mandible. This wire was gradually upgraded to a 0.019 × 0.025” stainless steel wire. The en-masse retraction in the mandibular arch was achieved with the help of mini-implants (1.5 mm x 8 mm) till 1 mm of space remained on the both sides.

**Figure 4:**
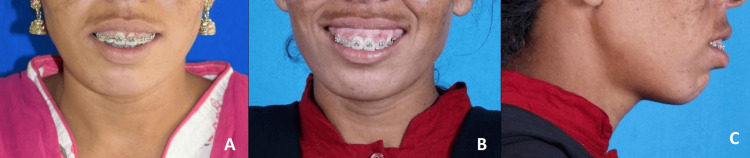
Pre-surgical extra-oral photographs. A: Frontal view; B: Frontal smiling view; C: Profile view

**Figure 5:**
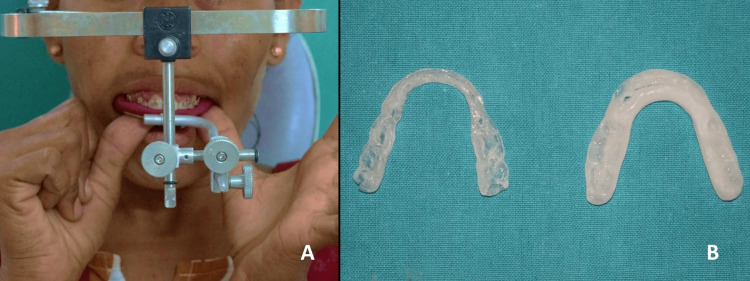
A: Face bow transfer; B: Intermediate and final splint fabrication

**Figure 6:**
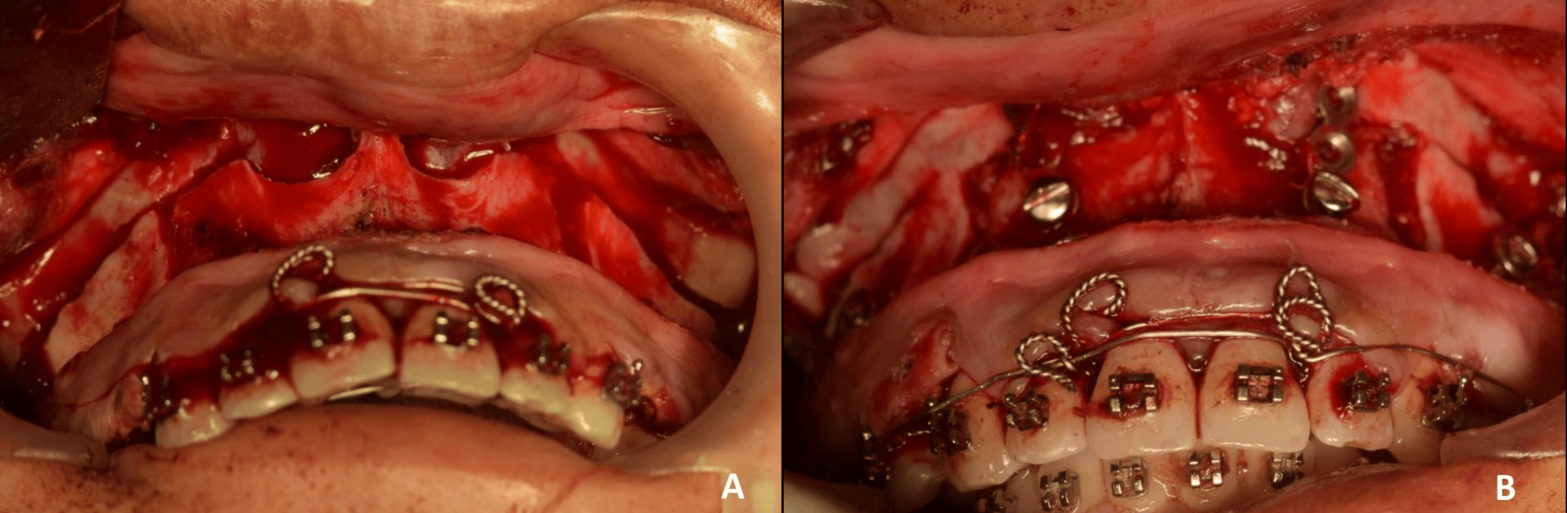
A: LeFort I osteotomy of 5 mm; B: Anterior segmental maxillary osteotomy of 6 mm with rigid fixation via titanium mini-plates

**Figure 7:**
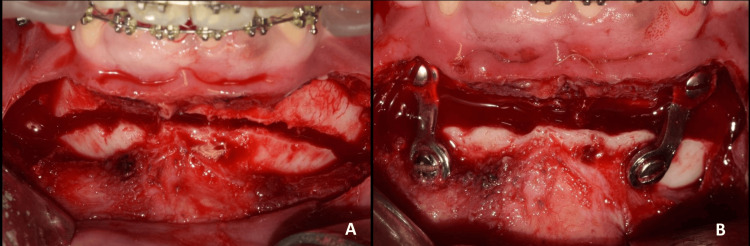
A: Advancement genioplasty by 5 mm; B: Rigid fixation via titanium mini-plates

After 11 months of presurgical orthodontics (Figure 4), the records were obtained, and the face bow record transfer was performed on Hanau’s semi-adjustable articulator (Figure 5). Following the mock surgery on the articulated models, an intermediate and a final acrylic surgical splint was constructed. LeFort I superior impaction of 5 mm was performed using a vestibular incision on the first maxillary molar with guiding holes in the superior nasal cavity and the antral side of the maxilla, followed by using a chisel and mallet separating the alveolar process from the palate to allow free movement of the alveolus providing a better maxillary impaction. It was followed by anterior segmental maxillary osteotomy setback of the maxilla by 6 mm after on-table extraction of maxillary first premolars bilaterally. An incision was made in the gingivobuccal sulcus, ensuring a minimum 1-cm tissue margin to facilitate easy closure, extended through the mentalis muscle down to the bone to carry out advancement genioplasty of 5 mm. Further, upper lip lengthening and alar cinching were carried out to address the short upper lip, VME, and protrusion of the maxilla, respectively (Figures 6, 7).

The surgery was carried out without complications, and the correction was stabilized using rigid two-point fixation via the following titanium mini-plates: 2 pre-shaped L plates of 12 mm, 0.6 mm thickness (4 holes) and 2 straight mini-plates of 12 mm (4 holes) for maxillary stabilization, and 2 straight mini-plates of 9 mm (2 holes with a gap) for stabilizing the advancement genioplasty. All the mini-plates were secured with self-drilling implants.

**Figure 8:**
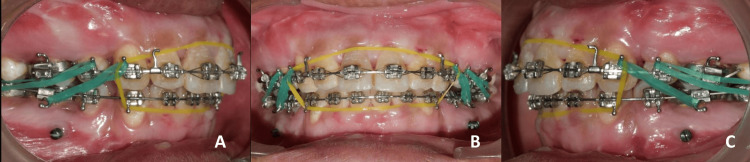
Post-surgical settling with short Class II elastics and anterior box elastics. A: Right occlusion view; B: Frontal View; C: Left occlusion view

**Figure 9:**
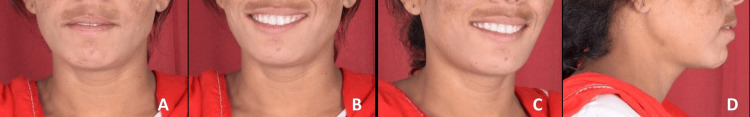
Post-surgical extra-oral photographs. A: Frontal view; B: Frontal smiling view; C: Oblique smiling view; D: Profile view

**Figure 10:**
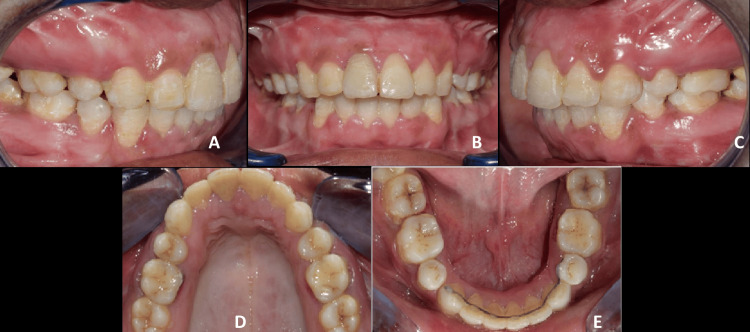
Post-surgical intra-oral photographs. A: Right occlusion view; B: Frontal view; C: Left occlusion view; D: Maxillary occlusal view; E: Mandibular occlusion view

Following orthognathic surgery, the patient was advised to rest for 15 days, leaving only to attend weekly postoperative follow-up appointments for eight weeks. After surgery, short Class II settling elastics (3/16”, 4.5 oz) and anterior box elastics (5/16”, 2 oz) were advised to the patient to guide the mandible into position after autorotation (Figure 8). The interdental spaces in the arches were consolidated, and the patient was advised to wear vertical settling elastics to achieve the final settling of the arches. Once the post-surgical finishing and detailing were completed, the appliance was removed (Figures 9, 10). The total treatment duration was 24 months. The treatment timeline was extended due to the patient's irregular attendance at follow-up appointments toward the end of the treatment period.

Treatment Results

**Figure 11:**
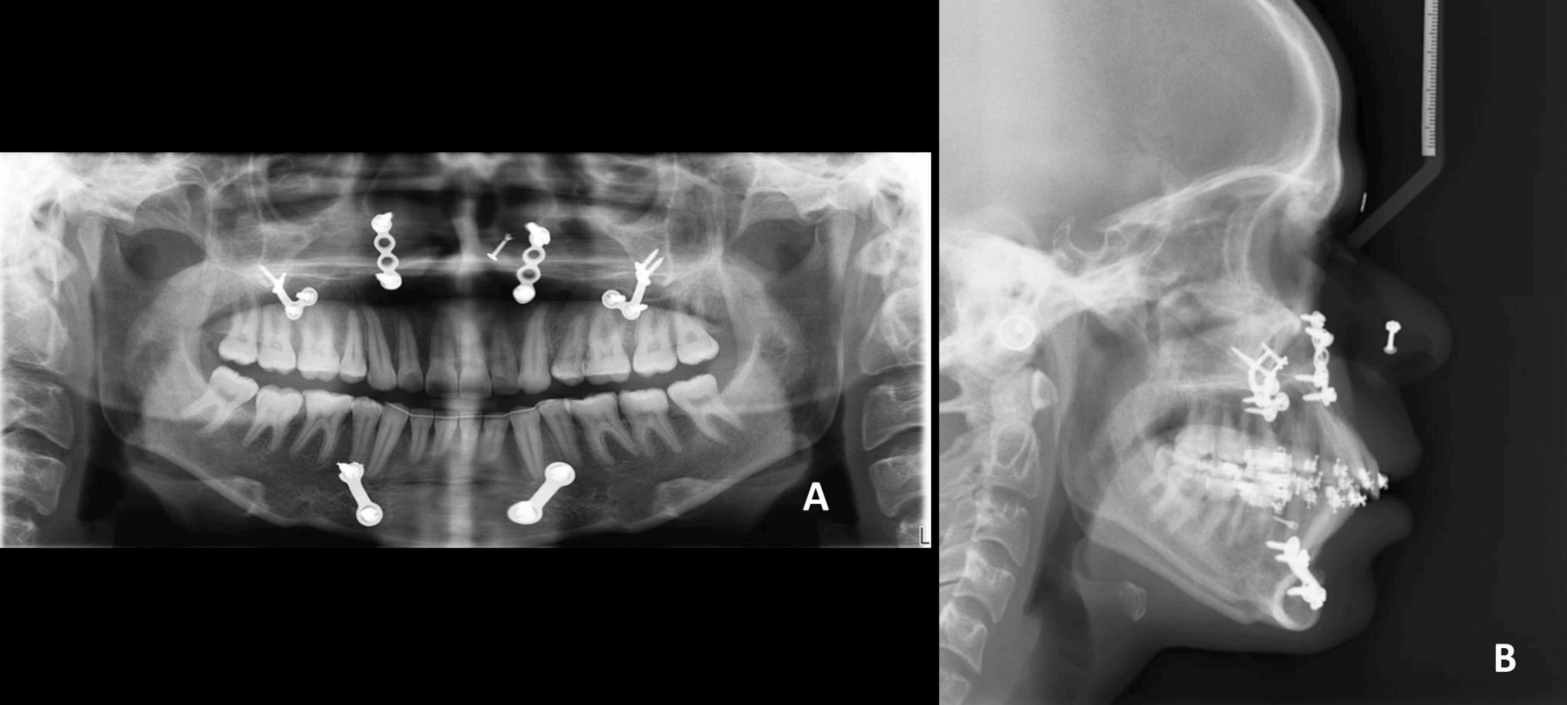
Post-treatment radiographs. A: Orthopantomogram; B: Lateral cephalogram

The post-treatment record analysis revealed a well-balanced maxillo-mandibular sagittal relationship with an ANB of 2° and Wits of 1 mm. An overall facial symmetry demonstrating proportionate facial thirds (maximum length: 52.5 mm, minimum length: 114 mm, LAFH: 63 mm), with a reduced gingival display, a consonant smile arc, and a satisfactory lip positioning (lower lip to E plane: 3 mm) was achieved (Figure 11; Table 2). 

**Table 2:** Post-treatment cephalometric summary

Variable	Post-treatment
Sagittal skeletal relationship	
SNA	84°
SNB	82°
ANB	2°
Wits appraisal	1 mm
Dental base relationship	
Upper incisor to NA	6 mm/28°
Lower incisor to NB	5 mm/30°
Upper incisor to SN plane	106°
Lower incisor to mandibular plane angle	94°
Dental relationship	
Interincisal angle	122°
Lower incisor to A-Pog line	6 mm
Overbite	2 mm
Over jet	3 mm
Vertical skeletal relationship	
Maxillary mandibular plane angle	30°
SN plane-mandibular plane	32°
Upper anterior face height	41 mm
Lower anterior face height	63 mm
Face height ratio	39.4:60.6
Jarabak ratio	66.6%
Maxillary length (ANS-PNS)	52.5 mm
Effective mandibular length	114 mm
Soft tissue	
Lower lip-E plane	3 mm
Nasolabial angle	94°

**Figure 12:**
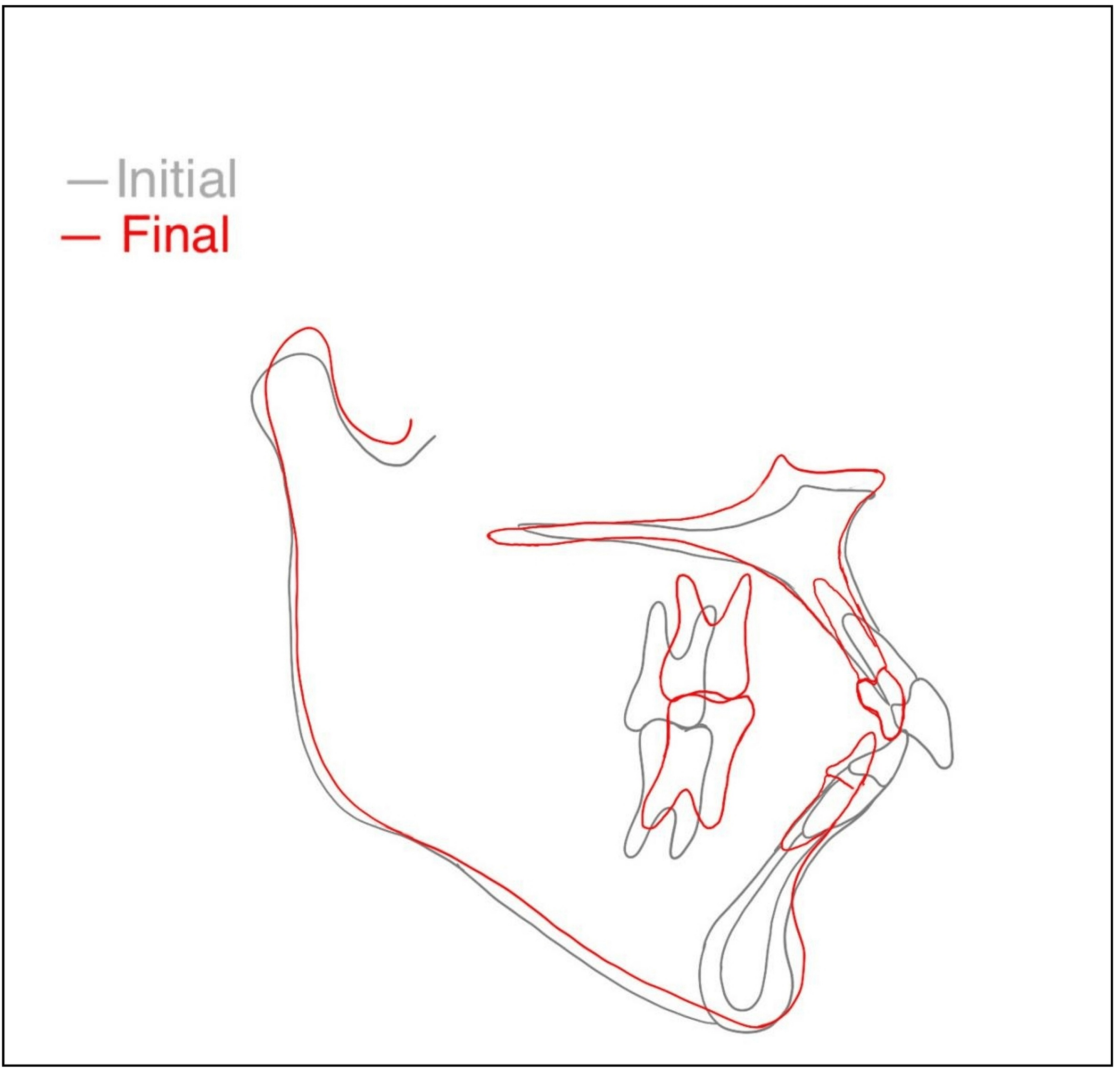
Superimposition of pre- and post-treatment outcome

After settling, the patient displayed a Class I molar, canine, and incisor relationships, coordinating upper and lower midlines, and harmonious arch forms. Cephalometric superimposition of pre- and post-treatment cephalograms (Figure 12) evidenced the amount of superior repositioning of the maxilla and the setback of the anterior segment. It also demonstrated the autorotation of the mandible and the advancement of the chin. The retention plan included a bonded lingual retainer in the lower arch and a removable retainer in the upper arch.

## Discussion

VME is characterized by excessive downward growth of the maxilla and surrounding dentoalveolar structures, which may affect the entire maxilla, or specifically in the posterior and/or anterior regions [6]. VME is associated with a long facial appearance, a gummy smile, and sometimes an open bite, all of which make the correction difficult.

A recommended treatment for excessive gingival display linked to VME involves reducing the vertical dimension of the maxilla through a LeFort I osteotomy [7]. Adjunct treatments, such as botulinum toxin injections or gingivectomy, do not yield satisfactory results. Orthopedic appliances, such as vertical pull chin cup or high pull headgear, are indicated in young growing patients as an alternative to orthognathic surgery in the future. However, their success is compliance dependent. Recent treatment modalities include TADs to correct the gummy smile [8].

Hence, the appropriate treatment modality, meticulous treatment planning, and execution are required to address the VME. Superior impaction of the maxilla is a chosen modality in cases where the vertical excess of the maxilla causes a downward and backward rotation of the mandible, leading to an increased tooth exposure at rest with incompetent lips.

The superior repositioning of the maxilla, combined with rigid internal fixation and genioplasty, is considered the most stable option, making the stability of superior impaction and setback of the maxilla as well as genioplasty excellent [9]. With the superior impaction of the maxilla, there is an alteration in the mandibular postural position in accordance with the new position of the maxilla, which in turn increases the occlusal forces, thus preventing the maxilla from relapsing downward.

The above-reported patient had a short upper lip with excessive incisor exposure at rest and increased gingival exposure during smile; thus, it was an ideal case for superior impaction of the maxilla. Though incisor exposure with 2 mm of gingival display on a smile is considered youthful and aesthetic, excessive gingival exposure affects the aesthetics negatively. In the present case, a conventional horseshoe LeFort I osteotomy was performed. When carrying out a conventional horseshoe procedure, it is important to assess the positional relationship between the palatal root apex of the maxillary molars and the maxillary sinus and nasal floor to prevent damaging the palatal root apex [10].

However, like any surgical procedure, the LeFort I osteotomy carries the risk of hemorrhagic complications. These can occur if the instrumentation is incorrect during the bone cutting or if the osteotome is inserted too high into the pterygopalatine fossa, potentially damaging the internal maxillary, sphenopalatine, and/or descending palatine arteries [11].

In order to address the setback of the anterior maxilla, the Epker’s-modified Cupar technique was used [12]. This technique helps preserve the palatal pedicle, facilitates the ease of internal fixation, and provides access to the nasal septal structures, thus preventing nasal septum buckling with superior repositioning of the maxilla.

The intermaxillary fixation was released after the completion of maxillary internal fixation, and the mandible is hinged with the condyles completely seated through the triangular finger formation, such that the mandible rotates upward to fit into the final maxillary splint.

The nasal cinch suture was placed, as it provides a suitable repositioning of the soft tissues and minimizes the postoperative widening of nasal base during LeFort I osteotomy [13]. In order to compensate for the lip shortening, thinning, and decreased vermillion show due to the typical maxillary repositioning, the V-Y closure was performed to counter the undesirable changes [14].

Clinically, an overall improvement in facial appearance was achieved, contributing significantly to the patient’s self-esteem.

## Conclusions

Careful diagnosis to identify the problem and proper treatment planning are of utmost importance when opting for surgical orthodontics in a patient. Surgical orthodontics offers an aesthetic improvement to a greater extent by harmonizing facial proportions. However, a good co-ordination is required between the orthodontist and the maxillofacial surgeon.
